# Orange and Sunlight: A Recipe for Blisters

**Published:** 2017-05

**Authors:** Stephanie Au, Ali Yousif, Suresh Anandan

**Affiliations:** 1 Department of Surgery, Glasgow Royal Infirmary, 84 Castle Street, Glasgow, UK;; 2 Department of Plastic Surgery, Wexham Park Hospital, Slough, UK

**Keywords:** Orange, Sunlight, Blister

**DEAR EDITOR**

Lime disease or phytophotodermatitis is a phototoxic inflammatory cutaneous eruption that occurs when skin is exposed to furanocoumarin-containing plants and sunlight. The presentations range from mild erythema to acute blisters or bullae.^1^ Common causative plants include lime, lemons, figs, parsnips and celery. The diagnosis is clinical and relies on meticulous review of exposure history, correlating the pattern of skin lesions with area of contact and excluding other causes of photodermatitis. Though the condition is not rare surgeons may encounter such cases and knowledge about the pathogenesis helps in the differential diagnosis.^1^^,^^2^

A 40 year-old man was referred to the Plastic Surgery Unit with an extensive rash involving both arms and legs. It developed over the course of two days, initially started with painful patches of erythema, which later developed into well demarcated, irregularly shaped plaques with clusters of vesicles and bullae (Figures 1 and 2). The distribution of the rash did not follow dermatomes. He was systemically well with no fever or joint pain. The rest of the physical examination was unremarkable. He had no significant past medical or dermatological history and was not on any regular medications. He was out in his garden for quite sometime but did not come in contact with any plants or insects. 

**Fig. 1:**
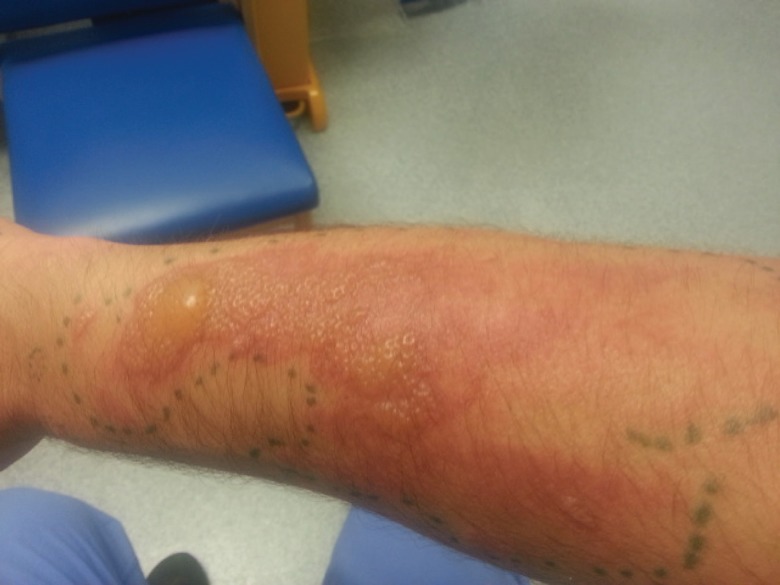
Appearance of skin lesion on day of presentation

**Fig. 2:**
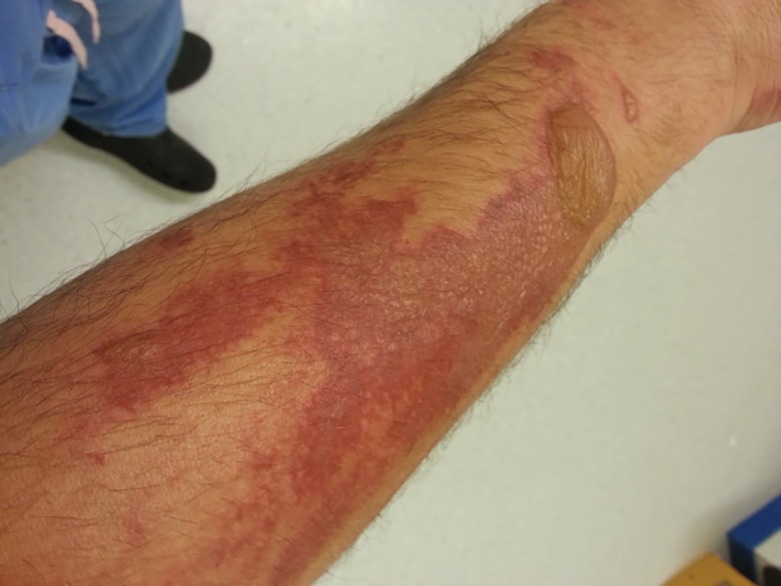
Appearance of skin lesion 2 days after presentation

He had no recent exposure to fragrances, perfumes or chemicals. However, on further questioning about any contact, he remembered that while squeezing oranges inside his house, he had accidentally splashed orange juice onto his hands, arms and legs, which he ignored and did not wash off. He then went straight to the garden afterwards. He realised that the rashes were in the areas the juice had come in contact with his body. Due to the history of exposure to orange juice and sunlight, the diagnosis of Lime disease or phytophotodermatitis was made. 

The patient was treated with two types of topical paraffin cream (Aquamax cream and Cetraben cream). The condition was explained to him and reassurance given. He was advised sun protection and to avoid photosensitizing agents to prevent future episodes. On follow up 8 days later, the erythema in the area had reduced. Vesicles and bullae had resolved and exfoliation had occurred (Figure 3). There was development of hyperpigmentation, which resolved over several months.

**Fig. 3:**
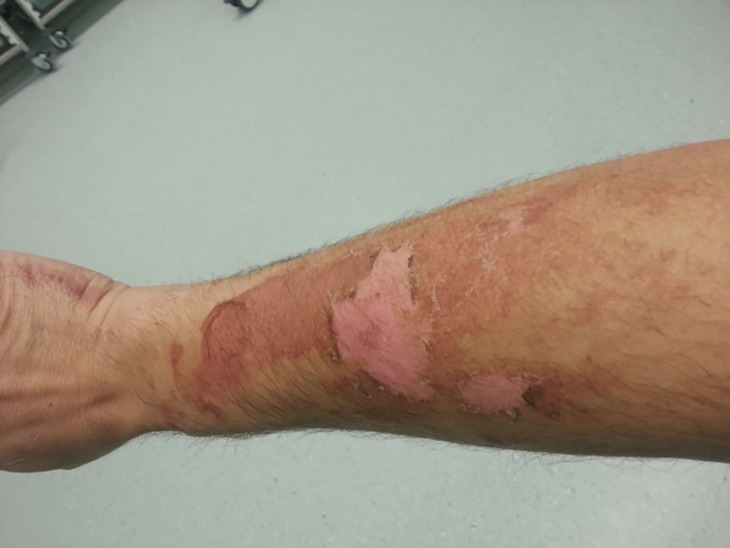
Appearance of skin lesion 8 days after presentation

Phytophotodermatitis is a phototoxic skin eruption due to contact with furocoumarins (psoralens), a compound found in plants, and subsequent exposure to long wavelength ultraviolet A (UVA) radiation. Common plants that contain furocuramins and found to be associated with phytophotodermatitis include those from the Rutaceae family (eg. lemon, lime), Umbelliferae family (eg. celery, parsnip, carrot), and the Moraceae family (e.g. fig).^3^ Orange is also a member of the Rutaceae family and this is the first case report in literature that reports the association between orange and phytophotodermatitis.

Such phototoxic reaction is non-immunological and does not require prior sensitization. It occurs via two mechanisms. The first mechanism involves photo-activated psoralens binding to pyrimidine bases of DNA and RNA covalently. The second mechanism involves psoralen-sensitized production of free radicals. These mechanisms results in cell membrane damage, leading to erythema, edema and formation of vesicles and bulle; and increase in proliferation of melanocytes and melanin production.^4^

The clinical manifestation of phytophotodermatitis varies from erythema to formation of vesicles and bullae and usually peaks in 36-72 hours after exposure to psoralens and UVA. The most striking feature is the odd shape and distribution of skin lesions that corresponds to the area of psoralens contact. Indeed, in cases where the fruit juice trickled down the body, the pattern of erythema could be seen as linear and trickle-shaped.^5^ In some paediatric cases, the parents were mistakenly accused of child abuse because the lesions corresponded to finger and hand marks that resulted from parents holding the children after being in contact with photosensitizing agents.^6^

Systemic symptoms, such as fever, are rare but can occur in severe generalized cases. Hyperpigmentation due to increased melanin production persists for several months after the acute episode. Diagnosis of phytophotodermatitis is clinical and requires meticulous history taking regarding contact with plants containing photosensitizing agents and subsequently sunlight exposure and correlating the distribution of skin lesion to areas of exposure are critical in arriving at the diagnosis. Treatment depends on the extent of involvement and it is usually symptomatic with cold compress, local wound care, topical emollient and topical corticosteroid in mild cases. Severe cases may require systemic corticosteroid and admission to a burn unit.^7^

The patients should be educated that this is a self-limiting condition although post-inflammatory hyperpigmentation can last for several months. They should also be advised to avoid contact with plants that contain photosensitising compounds and subsequent sunlight exposure to prevent future episodes. Phytophotodermatitis is an inflammatory phototoxic cutaneous eruption triggered by contact with furocuramins-containing plants such as citrus fruits and figs and subsequent exposure to UVA. The manifestation ranges from erythema to formation of blisters and bullae and the distribution of skin lesions correspond to the areas of contact. Treatment is symptomatic in mild cases but systemic corticosteroid and burn unit admission may be indicated in extensive and severe cases. Meticulous history enquiring about contact with any agents and correlating distribution of skin lesion with areas of contact is key in making the diagnosis.

## CONFLICT OF INTEREST

The authors declare no conflict of interest.
